# Exploring the relationship between serum vitamin D and atherosclerosis in hemodialysis patients: a cross-sectional study

**DOI:** 10.3389/fneph.2026.1738155

**Published:** 2026-06-10

**Authors:** Ashraf Hassan Abd El Mobdy, Saeed Abdelwahab Saeed, Walid Ahmed Bichari, Omaira Ahmed Osman, Shereen Yousef Salah

**Affiliations:** 1 Internal Medicine Department, Faculty of Medicine, Ain Shams University, Cairo, Egypt; 2 Internal medicine Department, Ahmed Maher Teaching Hospital, Cairo, Egypt

**Keywords:** atherosclerosis, carotid intima-media thickness, end-stage renal disease, maintenance hemodialysis, vitamin D

## Abstract

**Objective:**

To check if there is a link between good vitamin D levels in the blood and subclinical atherosclerosis in people on hemodialysis.

**Methods:**

Ninety end-stage renal disease patients undergoing regular hemodialysis were included in a cross-sectional study. Based on carotid intima-media thickness (CIMT), patients were grouped into Group A (n = 44) with normal CIMT and Group B (n = 46) with increased CIMT. Clinical assessments, laboratory parameters (serum vitamin D, lipid profiles, and inflammatory markers), and CIMT were measured using carotid ultrasound. The researcher compared students’ t-tests with quantitative variables, while employing the Chi-square or Fisher’s exact test with qualitative variables.

**Results:**

A positive correlation was observed between serum vitamin D and calcium (p = 0.010) and triglycerides (p = 0.025). Serum vitamin D showed a negative correlation with weight, serum alkaline phosphatase, and urea. No significant differences were found between the two groups in terms of demographic factors, BMI, hemodialysis duration, diabetes prevalence, or vitamin D supplementation. Group B exhibited elevated serum calcium levels (p = 0.008). Multivariate analysis found weight as an independent predictor of low vitamin D levels (p = 0.016).

**Conclusion:**

Serum calcium levels were significantly associated with CIMT; however, no correlation was seen between vitamin D levels and CIMT.

**Trial registration:**

registered at clinicaltrials.gov (ID: NCT07200804).

## 1 Introduction

Hemodialysis patients with End-Stage Renal Disease (ESRD) face a significantly elevated risk of cardiovascular disease (CVD) due to a complex interplay of factors. A 30-year-old HD patient faces a cardiovascular mortality risk equivalent to that of an 80-year-old in the general population, emphasizing the accelerated ageing of the cardiovascular system in ESRD (1). Pre-existing conditions like diabetes and hypertension, common causes of ESRD, also serve as traditional CVD risk factors (2). High uric acid in ESRD leads to harmful substance accumulation, persistent inflammation, increased oxidants, and excess body fluid (3). CKD-MBD disrupts bone and mineral metabolism, leading to vascular calcification that stiffens blood vessels and elevates cardiovascular risk (4). Once seen as passive, vascular calcification is now known to be an active, progressive disorder with significant clinical implications (5, 6). Anemia, another ESRD complication, increases cardiac load (7).

Hemodialysis introduces physiological challenges, including rapid fluid shifts and electrolyte imbalances, which can cause arrhythmias and hemodynamic instability. These disturbances arise from rapid volume depletion, autonomic dysfunction, and decreased vascular tone (8). The procedure may also cause swelling and blood clots, making coronary artery disease the leading cause of CVD in hemodialysis patients, who are at greater risk of sudden heart attack (9, 10).

The Malnutrition-Inflammation-Atherosclerosis (MIA) syndrome, prevalent in ESRD, significantly contributes to morbidity and mortality. This syndrome links malnutrition, systemic inflammation, and accelerated atherosclerosis—the primary cause of death in dialysis patients (11). In atherosclerosis, signs include thickened carotid walls, abnormal cholesterol levels, and arterial plaques.

CIMT, a surrogate marker for atherosclerosis, reflects generalized vascular changes. Early detection of increased CIMT can guide preventive strategies (12). Vitamin D may reduce inflammation and modulate immunity, lowering hypertension and dyslipidemia risks and possibly reversing vascular calcification (13). However, excessive supplementation can cause hypercalcemia (14). A research gap remains regarding vitamin D’s direct and indirect vascular effects.

Thus, this study aimed to determine the correlation between serum vitamin D level and subclinical atherosclerosis in hemodialysis patients.

## 2 Patients and methods

The cross-sectional study was carried out on 90 patients with ESRD undergoing hemodialysis at Ain Shams University or the National Institute of Nephrology and Urology. The study was approved by the Institutional Ethics Committee of the Faculty of Medicine, Ain Shams University, with approval number FMASU MSO 2/2025, dated and registered at clinicaltrials.gov (ID: NCT07200804).

Patients were recruited using a consecutive sampling method, in which all eligible patients undergoing maintenance hemodialysis at the study centers during the study period (from June 2021 to December 2021) were invited to participate.

Patients with ESRD who had been on maintenance hemodialysis for at least 6 months, aged 18 years, and receiving three weekly dialysis sessions with bicarbonate-based dialysate and anticoagulants were included in this study.

Patients with active infections, malignancies, autoimmune diseases, pregnancy, or pre-existing cardiovascular conditions were excluded. Pre-existing cardiovascular conditions were operationally defined as a documented history of coronary artery disease (including prior myocardial infarction, coronary revascularization, or angiographically proven stenosis), heart failure, cerebrovascular stroke or transient ischemic attack, or peripheral arterial disease diagnosed by a physician prior to study enrollment.

Everyone who participated in the study agreed to join after being informed of all the details of the experiment. Based on their CIMT, the patients were divided into two groups: Group A (n = 44) with normal CIMT (<0.8 mm) and Group B (n = 46) with increased CIMT (>0.8 mm).

Collect data on demographics (age, sex, comorbidities), dialysis duration, medications, and lifestyle factors (e.g., smoking, physical activity). About midweek, participants in the study underwent dialysis sessions. Blood samples for lipid profiles, urea, calcium, phosphorus, and hs-CRP were collected after an overnight fast of at least 8-12 hours to avoid postprandial fluctuations in lipid and inflammatory marker levels. Pre-dialysis blood samples were collected in the morning just before the patient’s dialysis session to reflect baseline biochemical levels without the influence of dialysis removal. For lipid profiles and 25(OH)D (vitamin D), blood was collected on non-dialysis days to avoid any immediate effects of dialysis on biomarkers. Blood was centrifuged promptly after collection to separate serum, which was stored at -80 °C for later analysis.

Blood levels of urea (mg/dL), calcium (mg/dL), phosphorus (mg/dL), alkaline phosphatase (U/L), and parathyroid hormone (pg/mL) were measured. Levels of HDL (mg/dL), triglycerides(mg/dL), and total cholesterol (mg/dL) were checked in the patient using an Abbott Aeroset autoanalyser with original kits (Abbott Laboratories, Abbott Park, Illinois, USA). Low-density lipoprotein cholesterol levels (LDL-C) (mg/dL) were calculated using the Friedewald equation. A radioimmunoassay procedure was used to measure 25(OH)D (DiaSorin, Stillwater, MN), Sufficient levels were considered > 30 ng/mL, and values between 20-30 ng/mL were considered insufficient. High-sensitivity C-reactive protein (hs-CRP) level was determined by the immunoturbidimetric method (Abbott Aeroset 1600, Abbott reagents, Germany).

Patients were classified according to their dietary intake of vitamin D, assessed through the Food Frequency Questionnaire (FFQ) and 24-hour dietary recall. This assessment was used to estimate long-term dietary habits and typical vitamin D-rich food consumption (e.g., fortified dairy, fatty fish). Daily dose of nutritional vitamin D was not strictly controlled but assessed through diet and supplements prescribed to patients.

Active vitamin D analogs (calcitriol or paricalcitol) were used as part of the treatment regimen for some patients, particularly those with secondary hyperparathyroidism or low serum calcium levels.

The dose and timing of active vitamin D analogs were individualized based on the patient’s serum calcium and parathyroid hormone (PTH) levels. Typical doses ranged from 0.25 µg to 2 µg of calcitriol or equivalent in paricalcitol per dialysis session, administered orally or intravenously after the dialysis session. The duration of therapy was consistent with clinical practice guidelines, which suggest that patients with ESRD often receive vitamin D supplementation throughout their dialysis treatment period.

### 2.1 Measurement of carotid intima-media thickness

CIMT was used as a validated and widely accepted surrogate marker of subclinical atherosclerosis. Samples were collected by measuring CIMT with a Mindray Real-time ultrasound scanner (DC-6 Doppler machine) and a 7.5 MHz probe. The measurement was taken at three different sites, each 1 cm proximal to the carotid bulb. The average of three values was taken to determine the final value. If CIMT > 0.8 mm, it was defined as thickened. The measurements adhered to the Mannheim CIMT Consensus (2004–2006) (12).

Intra-assay coefficients of variation (CV) and inter-assay coefficient of variation (CV) were calculated for all biochemical parameters to ensure the precision and consistency of the results across different tests. This data confirms that the results obtained are accurate and reliable, thereby enhancing the credibility of the study.

### 2.2 Sample size calculation

The sample size calculation was done by MedCalc Software Ltd v. 20 with 80% power, 5% confidence limit, and a correlation coefficient between Vitamin D and carotid intima-media thickness was -0.38 according to a previous study (15). Six cases were added to overcome dropout. Therefore, 90 patients were recruited for the study.

### 2.3 Statistical analysis

Statistical analysis was done by SPSS v26 (IBM Inc., Chicago, IL, USA). The Shapiro-Wilk test and histograms were used to assess the normality of the data distribution. Quantitative parametric variables were presented as mean ± standard deviation (SD) and compared between the two groups using an unpaired Student’s t-test. Quantitative nonparametric data were presented as median and interquartile range (IQR) and analyzed using the Mann-Whitney test. Qualitative variables were presented as frequencies and percentages (%) and analyzed using the Chi-square test or Fisher’s exact test, as appropriate. A two-tailed P value < 0.05 was considered statistically significant. Correlation between various variables was done using Pearson moment correlation equation for linear relation of normally distributed variables. Multivariate regression was also used to estimate the relationship between a dependent variable and more independent variables.

## 3 Results

In this study, 97 patients were assessed for eligibility. Five patients did not meet the inclusion criteria (three due to pre-existing cardiovascular disease and two due to dialysis duration <6 months), and two patients declined participation. Therefore, 90 patients were included in the final analysis. Based on their CIMT measurements, patients were divided into two groups: Group A (n = 44) with normal CIMT (<0.8 mm) and Group B (n = 46) with increased CIMT (≥0.8 mm).

**Table 1** summarizes the demographic data for the participants. No significant differences were observed between Group A and Group B in terms of age, gender, weight, BMI, waist circumference, physical activity level, sunlight exposure, smoking, lung disease, duration of hemodialysis, the urea reduction ratio (URR), osteocalcin, use of vitamin D supplements, FFQ, and FFQ (24-hour dietary recall) were also not significant (P> 0.05).

**Table 1:** Demographic data of studied groups.

Demographic data			Group A (No. = 44)	Group B (No. = 46)	Test value	P-value	95%CI
Age			54.18 ± 6.31	54.49 ± 5.41	t = 0.247	0.802	-1.25 - 0.63
Sex		Female	18 (40.9%)	17 (37.0%)	X2 = 0.148	0.701	-–
		Male	26 (59.1%)	29 (63.0%)			
Weight (kg)			76.76 ± 6.87	78.1 ± 7.01	t = 0.917	0.362	-3.09 -0.41
BMI (kg/m^2^)			26.04 ± 4.1	26.68 ± 3.87	t = 0.761	0.448	-1.92 - 0.64
Waist circumference (cm)			87.64 ± 9.3	90.37 ± 9.11	t=1.41	0.162	-6.13 - 0.67
Physical activity level (min/week)			165.32 ± 15.85	157.11 ± 22.86	t=1.97	0.052	-0.03 -16.45
Sunlight Exposure (min/day)			46.84 ± 12.52	50.43 ± 11.2	t=1.44	0.154	-8.58 -1.41
Smoking		No	11 (25%)	12 (26.09%)	X2 = 0.064	0.954	-–
		Yes	33 (75%)	34 (73.91%)			
DM		No	28 (63.6%)	29 (63.0%)	X2 = 0.003	0.953	-–
		Yes	16 (36.4%)	17 (37.0%)			
Lung disease		No	42 (95.5%)	44 (95.7%)	X2 = 0.002	0.964	-–
		Yes	2 (4.5%)	2 (4.3%)			
Dialysis duration (months)			32.25 ± 12.33	33.02 ± 9.77	t = -0.330	0.742	-4.23 - 2.68
Dialysis Adequacy (URR) (%)			76.14 ± 6.23	74.87 ± 7.26	t=0.89	0.372	-1.99 - 4.53
Osteocalcin (ng/mL)			17.68 ± 3.48	16.83 ± 3.2	t=1.20	0.223	-0.60 - 2.30
Use of vitamin D			27 (61.4%)	26 (56.5%)	X2 = 0.218	0.641	-–
FFQ	No		25 (56.82%)	29 (63.04%)	X2 = 0.149	0.895	-–
	Yes		19 (43.18%)	17 (36.96%)			
FFQ (24-hour dietary recall) (IU/day)			396.84 ± 42.26	393.45 ± 34.63	t=0.417	0.677	-6.98 -13.77

Date are presented mean (± SD) or frequency (%). t, independent t-test; X2, chi-square test; URR, Urea reduction ratio; BMI, body mass index; DM, diabetes mellitus. FFQ, Vitamin D-rich food intake. P value for significance. CI, Confidence interval.

Serum calcium, CRP titer, LDL cholesterol, HDL cholesterol, triglyceride, total cholesterol, and parathyroid hormone showed no significant differences between the groups. Serum urea, serum phosphorus and serum alkaline phosphatase were significantly higher in group B than in group A (P<0.05). serum 25(OH) Vitamin D were significantly lower in group B than in group A (P<0.05). **Table 2**.

**Table 2:** Laboratory data of studied cases of studied cases.

Data	Group A	Group B	Test of significance	P value	95%CI
**Serum urea (mg/dL)**	35.6± 17.8	63.9± 20.8	t = 6.92	**< 0.001**	(-35.7, -20.9)
**Serum calcium (mg/dL)**	9.1± 0.6	9.2 ± 0.9	t= 0.617	0.538	(-0.6, 0.4)
**Serum phosphorus (mg/dL)**	3.7± 1.0	4.9 ± 1.2	t=5.14	**< 0.001**	(-1.5, -0.9)
**Serum alkaline phosphatase (mg/dL)**	212.85 ± 26.31	338.25 ± 24.10	t=23.59	**< 0.001**	(-140.2, -110.6)
**CRP titer (mg/dL)**	0.2 ± 0.4	0.4± 0.6	t=1.861	0.067	(-0.45, 0.05)
**LDL Cholesterol (mg/dL)**	93.2± 26.1	94.6± 27.5	t=0.247	0.805	(-10.4, 7.6)
**HDL Cholesterol (mg/dL)**	34.5 ± 9.2	35.0± 10.6	t=0.238	0.812	(-4.1, 3.1)
**Triglyceride (mg/dL)**	140.1 ± 72.2	141.7 ± 78.5	t=0.1	0.920	(-11.8, 8.6)
Total cholesterol (mg/dL)	160.5 ± 36.2	159.4 ± 37.7	t=0.141	0.888	(-9.2, 11.4)
Serum 25(OH) Vitamin D (ng/ml)	13.9 ± 7.2	10.6 ± 6.3	t=2.31	**0.022**	(0.5, 6.1)
Parathyroid hormone (pg/dl)	138.43 ± 10.83	136.09 ± 7.76	t=1.16	0.246	(-1.2, 5.9)

T, Two-Sample Independent; t, Test. P value for significance (* Significant P value <0.05). CI, Confidence interval.

**Figure 1:**
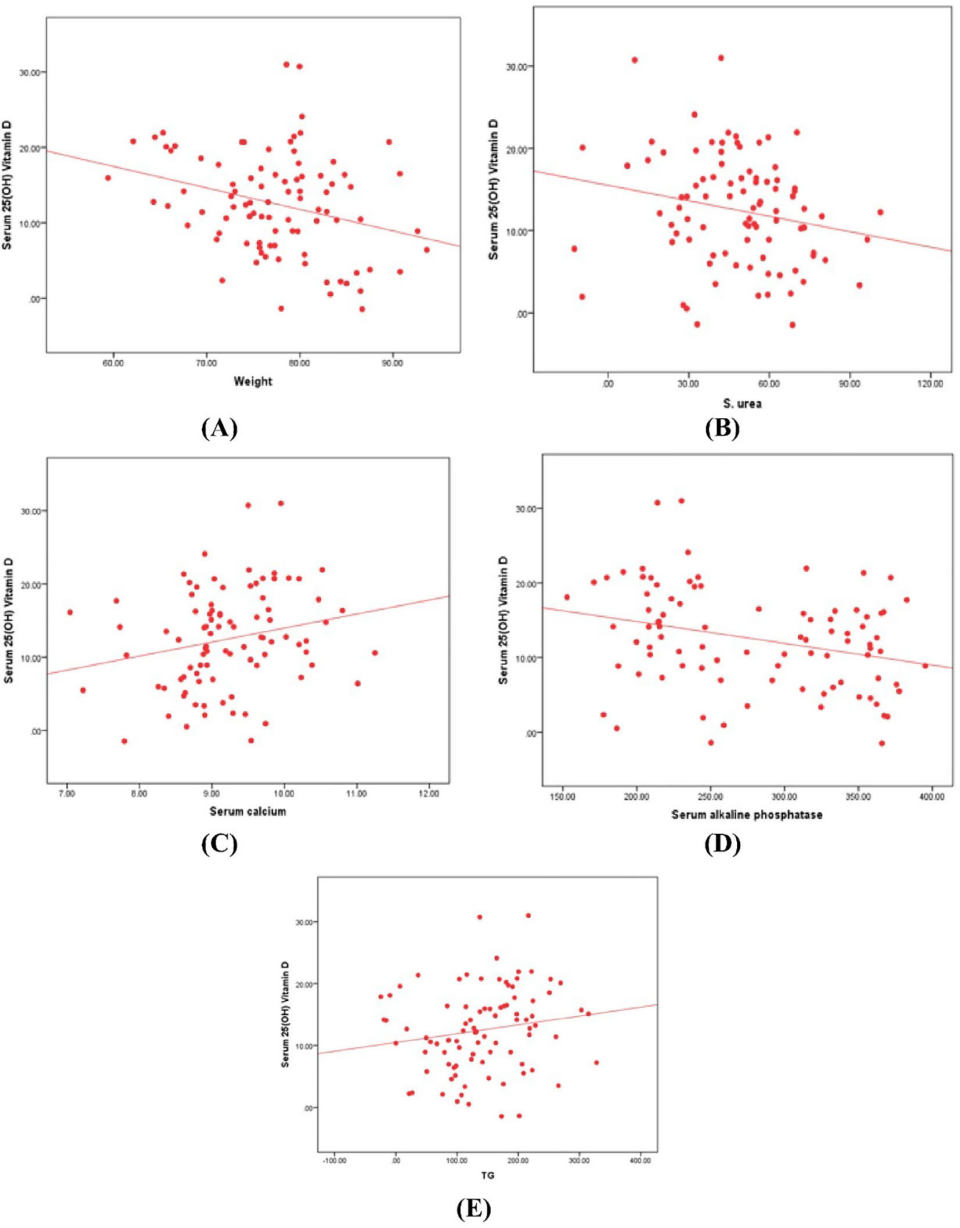
Correlation between Serum Vitamin D and Other Parameters. **(A)** Negative correlation between serum vitamin D and weight. **(B)** Negative correlation between serum vitamin D and S. urea. **(C)** Negative correlation between serum vitamin D and serum alkaline phosphatase enzyme. **(D)** Positive correlation between serum vitamin D and serum calcium, **(E)** Positive correlation between serum vitamin D and TG.

The correlations between vitamin D and other parameters were determined. There are obvious positive correlations between vitamin D levels and serum Ca and TG (p =0.010, p =0.025, respectively), and negative associations between vitamin D and weight, alkaline phosphatase level, and serum urea (p = 0.011, p = 0.003, p =0.039, respectively). Vit. D showed an insignificant association with age, BMI, URR, or dialysis duration. **Table 3** and **Figure 1**.

**Table 3:** Correlation between serum vitamin D and other parameters.

Study parameters	Serum Vitamin D (ng/ml)	
	r	P-value
Weight (kg)	-0.267*	0.011
BMI (kg/m^2^)	-0.024	0.821
Duration of dialysis (months)	-0.165	0.120
URR	0.097	0.365
Serum urea (mg/dL)	-0.219*	0.039
Serum calcium (mg/dL)	0.270*	0.010
Serum phosphorus (mg/dL)	-0.162	0.128
Serum alkaline phosphatase (mg/dL)	-0.305**	0.003
CRP titer (mg/dL)	-0.134	0.208
LDL (mg/dL)	0.131	0.219
HDL (mg/dL)	0.007	0.945
Serum Triglycerides (mg/dL)	0.236*	0.025
Serum cholesterol (mg/dL)	0.038	0.723
CIMT (mm)	-0.165	0.121

*P-value > 0.05: non-significant; P-value < 0.05: significant; P-value < 0.01: highly significant; r, Spearman correlation coefficient; URR, urea reduction ratio; BMI, body mass index; CRP, C-reactive protein; LDL, Low-density lipoprotein, HDL, High-density lipoprotein, CIMT, Carotid intima-media thickness.

** Highly Significant P <0.001.

The multivariate linear regression analysis showed insignificant effects between factors associated with serum CIMT (p > 0.05). **Table 4**.

**Table 4:** Multivariate linear regression analysis to factors associated with serum CIMT.

	Coefficient	Std. Error	t	P
Age	0.0023	0.002	0.8860	0.378
Sex	0.0250	0.04370	0.5780	0.56
Weight	0.00030	0.00200	0.1790	0.858
BMI	0.00190	0.00350	0.5550	0.580
DM	-0.0077	0.036	-0.2140	0.831
CRP titer	-0.0600	0.037	-1.5940	0.114
Smoking	-0.0240	0.042	-0.5820	0.562
Serum calcium	0.0490	0.02731	0.8160	0.073
Serum phosphorus	0.014	0.0145	0.9740	0.333
Serum 25(OH) Vitamin D	-0.003	0.0024	-1.6150	0.110

## 4 Discussion

It aimed to determine whether subjects on hemodialysis with low vitamin D levels also exhibit greater subclinical atherosclerosis, as indicated by CIMT thickness. The blood vitamin D levels did not correlate with CIMT results. These ideas were confirmed by Park et al., who investigated how vitamin D levels affect the risk of heart disease in ESRD patients. They found that serum vitamin D concentration was not a significant predictor of blood pressure or arterial stiffness in non-dialysis ESRD and HD patients (16, 17).

However, we observed significant correlations between serum vitamin D levels and several biochemical markers, including positive associations with serum calcium and triglycerides, and negative associations with weight, alkaline phosphatase (ALP), and serum urea. Mechanistically, vitamin D enhances intestinal calcium absorption and regulates parathyroid hormone (PTH) secretion, thereby maintaining calcium-phosphate balance and influencing ALP through bone turnover and mineralization. Its effect on triglycerides may involve modulation of lipid metabolism in hepatic and adipose tissues, as well as improvements in insulin sensitivity and inflammatory pathways (18). Gupta and Kumar supported the above conclusions in their study (19). It appears that vitamin D has a non-direct influence on atherosclerosis. The results declared that dialysis patients did not experience a direct correlation between vitamin D levels and signs of heart disease. Therefore, we did not find a strong association between people’s vitamin D levels and the thickness of their inner arterial walls. The findings of a study conducted by Junior in 2018 were consistent with those mentioned earlier, revealing no significant relationship between vitamin D and CIMT (20). On the other hand, Hu’s 2024 study found that serum vitamin D levels were negatively correlated with triglycerides (21). These differences may arise from variations in study populations or sample sizes.

While the above studies found no correlation or a negative correlation between serum vitamin D and atherosclerosis, a few studies have shown that vitamin D is protective for cardiovascular health. A systematic review and meta-analysis by Säidifard et al. (23) found that vitamin D is protective against atherosclerosis. The results clearly stated “our findings show that serum vitamin D is inversely associated with CIMT, and vitamin D supplementation may reduce CIMT.

Although our study did not show a correlation between serum vitamin D and cardiovascular health, some studies support a protective role for serum vitamin D. One such study stated, “vitamin D supplementation may be useful in hypercholesterolemia patients with vitamin D insufficiency who are at high risk of cardiovascular diseases” (22). A study by Canhos also supported these findings, concluding that “an association between serum vitamin D levels and mortality in hemodialysis patients” exists (23). Although our study did not show a link between serum vitamin D and atherosclerosis, other studies have. In the same year, Khanolkar outlined how Vitamin D can decrease inflammation, improve blood vessel function, and reduce vascular smooth muscle cell growth and bone thickness. These mechanisms suggest indirect pathways by which vitamin D may influence CIMT via reduced vascular inflammation, improved endothelial function, and modulation of calcium-phosphate homeostasis (24).

It also influences calcium-phosphate homeostasis, which is particularly relevant in hemodialysis patients who are prone to vascular calcification due to mineral imbalance (25). In addition, our study correlated serum calcium and CIMT and supported the concept that mineral dysregulation contributes to vascular pathology in ESRD. This finding aligns with a 2019 study by Viegas. The study explains in detail that “since hypocalcemia stimulates parathyroid hormone (PTH) production and decreased levels of vitamin D result in continued secretion of PTH, an excessive rise in PTH release, often accompanied by parathyroid hyperplasia, is common in ESRD patients. A further decline follows this in kidney function, ESRD progression, and cardiovascular outcomes” (26).

Another role of vitamin D in affecting ALP levels is its regulation of bone breakdown and calcification of blood vessels via the CKD-MBD pathways. This was also demonstrated by a 2024 study by Yeung and coworkers, which found that Vitamin D may be associated with lipid metabolism (17). Our analysis also showed a positive association between serum vitamin D and triglycerides, consistent with a previous meta-analysis by Huang et al. (28), which reported a positive correlation between vitamin D and elevated triglyceride levels.

And this result aligns with findings from Parizadeh et al., who reported that “the study showed different beneficial effects of a normal level of vitamin D level or vitamin D following supplementation therapy” (27).

As discussed earlier, our study did not confirm a direct link between blood vitamin D levels and CIMT; however, it highlighted the potential importance of serum vitamin D status in treating hemodialysis patients. Ishtawi found that vitamin D plays a crucial role in reducing kidney complications in individuals with CKD (28). Routine screening for vitamin D deficiency and cautious supplementation may offer benefits in bone health, inflammation control, and metabolic balance. This agrees with Jorgensen. However, larger interventional studies are needed (29). Research by Vervloet in 2023 concluded that supplementing vitamin D at an adequate dose benefits CKD patients, but further research is still required to make stronger claims (30). However, given the lack of a clear causal relationship with atherosclerosis, clinical decisions regarding vitamin D supplementation should be individualized, and more robust evidence is needed to justify its use for cardiovascular protection.

The present study has several important strengths. First, it addresses a clinically relevant question in a high-risk population—patients with end-stage renal disease (ESRD) undergoing maintenance hemodialysis—who are disproportionately affected by cardiovascular morbidity and mortality. Second, we employed objective and validated markers of both serum vitamin D status and subclinical atherosclerosis, including serum 25(OH) vitamin D levels and carotid intima-media thickness (CIMT), measured according to the Mannheim consensus. Third, the use of a comprehensive biochemical panel allowed for a broader evaluation of mineral metabolism, lipid profile, and inflammatory status. Finally, patients were recruited consecutively, reducing potential selection bias and enhancing the internal validity of the study. Due to the cross-sectional study design, we were unable to establish cause-and-effect relationships between vitamin D levels and atherosclerosis. The study results cannot be generalized as they were conducted at a single center. Moreover, we did not adjust for potential confounders, such as parathyroid hormone (PTH) levels, calcium-phosphate product, and dialysis adequacy, which could have influenced the results. Despite efforts to control standard variables, residual confounding cannot be ruled out.

Researchers should examine groups with similar traits over time to determine if serum vitamin D is associated with a person’s risk of atherosclerosis. Randomized controlled trials should be conducted to investigate the effect of vitamin D on cardiovascular complications in people undergoing hemodialysis. They could study whether varying vitamin D forms, with a special emphasis on 1,25-dihydroxyvitamin D, are associated with the accumulation of calcium in blood vessels and inflammation.

This study concluded that no association was found between serum vitamin D levels and atherosclerosis, as assessed by CIMT, among hemodialysis patients. Even so, several metabolic parameters were associated with serum vitamin D, suggesting an indirect association with heart risk. The results indicate that multiple factors influence vascular function in ESRD, suggesting that further studies are needed to determine whether vitamin D can be beneficial in combating atherosclerosis and protecting these patients from related diseases.

Limitations and recommendations: Since only a few patients and one center were involved, the findings cannot be generalized to a larger scale. Investigating the connection between vitamin D and cardiovascular health, as well as ESRD, in a broader and larger patient population is necessary to determine the most effective means of prevention. Despite the use of consecutive sampling, residual selection bias cannot be completely ruled out due to the study’s single-center design. As this was an observational cross-sectional study, confounding by indication related to vitamin D supplementation and mineral metabolism may have influenced the observed associations. Multivariate modelling can help provide a better understanding of how vitamin D supplementation affects vascular health outcomes. Conducting longitudinal studies would help examine how changes in vitamin D levels over time may contribute to the development of atherosclerosis. These findings could provide valuable insights for developing effective strategies to promptly identify and manage cardiovascular risk in patients with ESRD.

It is essential to consider individualized treatment approaches that incorporate both traditional and non-traditional risk factors, including vitamin D levels and calcium-phosphate balance. It may also be beneficial to implement targeted treatments to address these risk factors and help prevent or delay atherosclerosis progression, leading to better outcomes.

Future longitudinal and multicenter studies with larger sample sizes are recommended to clarify the causal relationship between serum vitamin D levels and subclinical atherosclerosis in hemodialysis patients. Randomized controlled trials are also needed to assess the vascular effects of vitamin D supplementation while adjusting for key confounders such as mineral metabolism and dialysis adequacy. Until more substantial evidence is available, vitamin D supplementation should be individualized and carefully monitored in this population.

## 5 Conclusion

Although the exact link between insufficient vitamin D levels and cardiovascular risk could not be proven, the study highlighted the most critical factors affecting heart disease in ESRD patients treated with hemodialysis. Additionally, a strong association was observed between serum calcium and CIMT, suggesting that weight is linked to both vitamin D and cardiovascular disease as an independent factor. This highlights the importance of considering unusual risk factors for heart health.

## Data Availability

The raw data supporting the conclusions of this article will be made available by the authors, without undue reservation.

## References

[1] Fong JMN Sia C-H See KC . Chronic kidney disease is no longer a ‘non-traditional’ cardiac risk factor: a call to action for cardiovascular-kidney-metabolic health. In: Medknow (2025). Wolters Kluwer Health. p. 122–4. 10.4103/singaporemedj.SMJ-2025-012 PMC11991072 40116056

[2] Hirsch D Lau B Kushwaha V Yong K . The controversies of coronary artery disease in end-stage kidney disease patients: a narrative review. Rev Cardiovasc Med. (2023) 24:181. doi:10.31083/j.rcm2406181 39077541 PMC11264163

[3] Stenvinkel P Carrero JJ Axelsson J Lindholm B Heimbürger O Massy Z . Emerging biomarkers for evaluating cardiovascular risk in the chronic kidney disease patient: how do new pieces fit into the uremic puzzle? Clin J Am Soc Nephrol. (2008) 3:505–21. doi:10.2215/cjn.03670807 18184879 PMC6631093

[4] Zaimi M Grapsa E . Current therapeutic approach of chronic kidney disease‐mineral and bone disorder. Ther Apheresis Dialysis. (2024) 28:671–89. doi:10.1111/1744-9987.14177 38898685

[5] Block GA Hulbert-Shearon TE Levin NW Port FK . Association of serum phosphorus and calcium x phosphate product with mortality risk in chronic hemodialysis patients: a national study. Am J Kidney Dis. (1998) 31:607–17. doi:10.1053/ajkd.1998.v31.pm9531176 9531176

[6] Mizobuchi M Towler D Slatopolsky E . Vascular calcification: the killer of patients with chronic kidney disease. J Am Soc Nephrol. (2009) 20:1453–64. doi:10.1681/asn.2008070692 19478096

[7] Xu C Tsihlis G Chau K Trinh K Rogers NM Julovi SM . Novel perspectives in chronic kidney disease-specific cardiovascular disease. Int J Mol Sci. (2024) 25:2658. doi:10.3390/ijms25052658 38473905 PMC10931927

[8] Jankowski J Floege J Fliser D Böhm M Marx N . Cardiovascular disease in chronic kidney disease: pathophysiological insights and therapeutic options. Circulation. (2021) 143:1157–72. doi:10.1161/circulationaha.120.050686 33720773 PMC7969169

[9] Neirynck N Vanholder R Schepers E Eloot S Pletinck A Glorieux G . An update on uremic toxins. Int Urol Nephrol. (2013) 45:139–50. doi:10.1007/s11255-012-0258-1 22893494

[10] Stevens PE Ahmed SB Carrero JJ Foster B Francis A Hall RK . KDIGO 2024 clinical practice guideline for the evaluation and management of chronic kidney disease. Kidney Int. (2024) 105:S117–314. doi:10.1016/j.kint.2023.10.018 38490803

[11] Mikami R Mizutani K Gohda T Matsuyama Y Gotoh H Nakagawa K . Malnutrition-inflammation-atherosclerosis (MIA) syndrome associates with periodontitis in end-stage renal disease patients undergoing hemodialysis: a cross-sectional study. Sci Rep. (2023) 13:11805. doi:10.1038/s41598-023-38959-0 37479734 PMC10361958

[12] Lawal OM Balogun MO Akintomide AO Ayoola OO Mene-Afejuku TO Ogunlade O . Carotid intima-media thickness: a surrogate marker for cardiovascular disease in chronic kidney disease patients. Clin Med Insights: Cardiol. (2019) 13:1179546819852941. doi:10.1177/1179546819852941 31258338 PMC6589967

[13] Bouderlique E Tang E Zaworski J Coudert A Bazin D Borondics F . Vitamin D and calcium supplementation accelerate vascular calcification in a model of pseudoxanthoma elasticum. Int J Mol Sci. (2022) 23:2302. doi:10.3390/ijms23042302 35216422 PMC8878394

[14] Mio K Haruhara K Shimizu A Oshiro K Kawai R Ikeda M . Hypercalcemia worsened after vitamin D supplementation in a sarcoidosis patient: a case report. Medicine. (2022) 101:e30883. doi:10.1097/md.0000000000030883 36221396 PMC9542661

[15] Fotoh DS Serag DM Badr IT Saif DS . Prevalence of subclinical carotid atherosclerosis and vitamin D deficiency in Egyptian ankylosing spondylitis patients. Arch Rheumatol. (2020) 35:335–42. doi:10.46497/ArchRheumatol.2020.7694 33458656 PMC7788658

[16] Park KM Jun HH Bae J Choi YB Yang DH Jeong HY . 25-hydroxyvitamin D levels was not associated with blood pressure and arterial stiffness in patients with chronic kidney disease. Electrolytes Blood Pressure. (2017) 15:27–36. doi:10.5049/ebp.2017.15.2.27 29399021 PMC5788812

[17] Yeung WCG Toussaint ND Lioufas N Hawley CM Pascoe EM Elder GJ . Vitamin D status and intermediate vascular and bone outcomes in chronic kidney disease: a secondary post hoc analysis of IMPROVE‐CKD. Internal Med J. (2024) 54:1960–9. doi:10.1111/imj.16516 39225105 PMC11610653

[18] Bhattarai HK Shrestha S Rokka K Shakya R . Vitamin D, calcium, parathyroid hormone, and sex steroids in bone health and effects of aging. J Osteoporos. (2020) 2020:9324505. doi:10.1155/2020/9324505 32612801 PMC7317615

[19] Gupta A Kumar S . Correlation of vitamin D level with its related biochemical parameters and impact of different treatment regimens on their correction. Int J Contemp Pediatr. (2020) 8:86–91. doi:10.18203/2349-3291.ijcp20205511

[20] Monteiro F Mandarino N Santos E Santos A Salgado J Brito D . Correlation between serum 25-hydroxyvitamin D levels and carotid intima-media thickness in a Brazilian population descended from African slaves. Braz J Med Biol Res. (2018) 51:e7185. doi:10.1590/1414-431x20177185 29490002 PMC5856431

[21] Hu T Zhang Y Chen Z Su J . Relationship between serum vitamin D levels and the atherogenic index of plasma: a study based on NHANES database 2011–2018. Front Nutr. (2024) 11:1468284. doi:10.3389/fnut.2024.1468284 39555194 PMC11566743

[22] Dibaba DT . Effect of vitamin D supplementation on serum lipid profiles: a systematic review and meta-analysis. Nutr Rev. (2019) 77:890–902. doi:10.1093/nutrit/nuz037 31407792

[23] da Silva Canhos MM De Oliveira RC Modelli de Andrade LG Caramori JCT Barretti P Martin LC . Association between vitamin D levels and mortality in hemodialysis patients: a cohort study. Renal Failure. (2020) 42:225–33. doi:10.1080/0886022x.2020.1735415 32126885 PMC7067165

[24] Młynarska E Lisińska W Hossa K Krupińska N Jakubowska P Rysz J . Vitamin D and chronic disorders: a review of metabolic and cardiovascular diseases. Pharmaceuticals. (2025) 18:1467. doi: 10.1093/ndt/gfae293 41155582 PMC12567385

[25] Khanolkar S Hirani S Mishra A Vardhan S Hirani S Prasad R . Exploring the role of vitamin D in atherosclerosis and its impact on cardiovascular events: a comprehensive review. Cureus. (2023) 15:5–10. doi:10.7759/cureus.42470 37637551 PMC10450567

[26] Viegas C Araújo N Marreiros C Simes D . The interplay between mineral metabolism, vascular calcification and inflammation in chronic kidney disease (CKD): challenging old concepts with new facts. Aging (Albany NY). (2019) 11:4274. doi:10.18632/aging.102046 31241466 PMC6628989

[27] Parizadeh SM Rezayi M Jafarzadeh-Esfehani R Avan A Ghazizadeh H Emadzadeh M . Association of vitamin D status with liver and kidney disease: a systematic review of clinical trials, and cross-sectional and cohort studies. Int J For Vitamin Nutr Res. (2020) 91(1-2):175–87. doi:10.1024/0300-9831/a000540 30816821

[28] Ishtawi S Jomaa D Nizar A Abdalla M Hamdan Z Nazzal Z . Vitamin D level, pain severity and quality of life among hemodialysis patients: a cross-sectional study. Sci Rep. (2023) 13:1182. doi:10.1038/s41598-022-25793-z 36681707 PMC9867695

[29] Jørgensen HS Vervloet M Cavalier E Bacchetta J de Borst MH Bover J . The role of nutritional vitamin D in chronic kidney disease–mineral and bone disorder in children and adults with chronic kidney disease, on dialysis, and after kidney transplantation—a European consensus statement. Nephrol Dialysis Transplant. (2025) 40:797–822. 10.1093/ndt/gfae293 PMC11960744 39875204

[30] Vervloet MG Hsu S de Boer IH . Vitamin D supplementation in people with chronic kidney disease. Kidney Int. (2023) 104:698–706. doi:10.1016/j.kint.2023.07.010 37541585

