# Supplementary material for: Exploring the relationship between serum vitamin D and atherosclerosis in hemodialysis patients: a cross-sectional study
Source: Front Nephrol. 2026 Jun 10;6:1738155. doi: 10.3389/fneph.2026.1738155 (PMC13291148; doi:10.3389/fneph.2026.1738155)
Supplement: Supplementary file 1 [file Table1.docx]

**Supplementary Table 1.** Laboratory data of studied cases (participants with an elevated CIMT)

| **Data** | **Group A** | **Group B** | **Test of Significance** | **P value** |
| --- | --- | --- | --- | --- |
| **Serum urea (mg/dL)** | 35.6 ± 17.8 | 63.9 ± 20.8 | t = 1.36 | 0.305 |
| **Serum calcium (mg/dL)** | 9.1 ± 0.6 | 9.2 ± 0.9 | t=2.25 | 0.008* |
| **Serum phosphorus (mg/dL)** | 3.7 ± 1.0 | 4.9 ± 1.2 | t=1.44 | 0.23 |
| **Serum alkaline phosphatase (mg/dL)** | 212.85±26.31 | 338.25±24.10 | t=1.19 | 0.56 |
| **CRP titer (mg/dL)** | 0.2±0.4 | 0.4 ± 0.6 | t=1.861 | 0.066 |
| **LDL Cholesterol (mg/dL)** | 93.2 ± 26.1 | 94.6 ± 27.5 | t=1.11 | 0.73 |
| **HDL Cholesterol (mg/dL)** | 34.5±9.2 | 35.0 ± 10.6 | t=1.3 | 0.35 |
| **Serum Triglycerides(mg/dL)** | 140.1±72.2 | 141.7±78.5 | t=1.18 | 0.58 |
| **Total cholesterol (mg/dL)** | 160.5±36.2 | 159.4±37.7 | t=1.08 | 0.78 |
| **Serum Vitamin D (ng/ml)** | 13.9±7.2 | 10.6±6.3 | t=1.3 | 0.37 |

*T = Two-Sample Independent t Test
